# Low short-term complication rates following acromioclavicular joint surgery: a large database study

**DOI:** 10.1016/j.xrrt.2025.100660

**Published:** 2026-01-02

**Authors:** Shahabeddin Yazdanpanah, Grayson M. Talaski, Matthew S. Smith, Braeden R. Gooch, Benjamin P. Cassidy, Andrew S. Cuthbert, Jennifer L. Vanderbeck

**Affiliations:** a College of Medicine, Northeast Ohio Medical University, Rootstown, OH, USA; b Department of Orthopedics and Rehabilitation, University of Iowa, Iowa City, IA, USA; c Department of Orthopaedic Surgery, Virginia Commonwealth University, Richmond, VA, USA

**Keywords:** Acromioclavicular, Acromioclavicular joint, AC joint, Outcomes, Database, NSQIP

## Abstract

**Background:**

Acromioclavicular (AC) joint injuries represent approximately 11% of all shoulder injuries and are managed surgically in severe cases via techniques such as hook-plating, button fixation, and graft-based reconstruction. While much of the existing literature on AC joint surgery points to relatively high rates of long-term complications and reoperations, short-term outcomes are not fully understood. Therefore, this study investigates short-term outcomes following AC joint surgery using a large database to provide comprehensive complication data and elucidate risk factors.

**Methods:**

The American College of Surgeons National Surgical Quality Improvement Program database was queried from 2010 to 2023. Patients undergoing surgical intervention for AC joint injuries were identified using Current Procedural Terminology 23550, 23552, and 21320, and their 30-day postoperative outcomes were retrieved. Patients with unknown or null values for demographic or complication metrics were excluded. Statistical analyses included multivariate odds-ratio (OR) logistic regression. Operative time threshold analysis was performed to identify the optimal time cut-point associated with increased complication risk.

**Results:**

A total of 13,117 patients underwent AC joint surgery (average age 49.6 ± 15.2 years; average body mass index 30.1 ± 6.44 kg/m^2^; 70.5% male). The overall adverse event rate was 2.7%: surgical site infection (1.2%) and return to operating room (1%) were among the most common. An average operating time of 85 ± 56 minutes was determined, and threshold analysis revealed a significant increase (*P* < .001) in complications for operations lasting longer than 148 minutes. Operative time (OR = 1.01), history of chronic obstructive pulmonary disease (OR = 2.47), steroids (OR = 3.16), dialysis (OR = 5.57), bleeding disorders (OR = 2.67), and type 1 diabetes (OR = 1.61) were all significant risk factors for complications.

**Conclusion:**

AC joint surgery demonstrated relatively low short-term complication rates; however, comorbidities such as type 1 diabetes and chronic obstructive pulmonary disease are linked to a higher risk of experiencing adverse events. Preoperative counseling is recommended for at-risk patients, and future studies should explore surgery-specific operative time and patient management to provide further insights and enhance surgical decision-making.

Acromioclavicular (AC) joint injuries are relatively common in adults, approximating 11% of all shoulder injuries.^37^ The incidence of AC injuries has risen by roughly 70% since the early 2000s according to some studies and has been documented at 3.1 per 100,000. The vast majority (>80%) of cases occur in males with a median age of around 30, particularly during sport or sports-related activities.^5^^,^^37^ Trauma to the joint from an axial load during adduction or landing on an outstretched arm are the most common mechanisms behind AC joint injury. The severity of such injuries are determined by Rockwood classification: types I and II are less severe and are often managed nonoperatively, types IV and V are more severe and indicate surgical treatment, and type III is managed both operatively and nonoperatively, as there is mixed literature on the most optimal treatment method.^5^^,^^22^^,^^32^^,^^35^^,^^36^^,^^42^

Surgical treatment for AC joint injuries includes a wide range of techniques, utilizing both arthroscopy and open reduction and internal fixation. Operative choice depends on injury severity, clinical findings, surgeon preference, and various patient-specific factors.^6^^,^^13^^,^^44^ While AC joint stabilization procedures often result in improved patient-reported outcome measures, there are grounds for concern in the literature regarding certain complications, including reconstructive and structural failures with loss of reduction, chronic pain, and/or shoulder instability.^6^^,^^11^^,^^35^ In addition, there are well-documented overall complication rates presumed to be due to surgical technique choice, delay to surgery, and select patient-specific factors like body mass index (BMI).^3^^,^^11^^,^^13^^,^^21^^,^^24^^,^^33^^,^^34^ In existing literature, complication rates vary, as both individual studies and larger reviews have reported ranges from 11% to 35% depending on surgical technique and patient cohort.^6^^,^^14^^,^^26^^,^^27^^,^^31^^,^^39^

Although there is a myriad of existing literature on AC joint injury outcomes, knowledge gaps exist in recent short-term understanding, particularly regarding risk factors for outcomes such as adverse events (AEs) and both targeted patient-specific and surgical contraindications. For example, Chen et al^6^ found that patients who underwent AC joint reconstruction more than 6 weeks after injury had 3.19 times greater odds of experiencing a complication at 6-month follow-up. In addition, Choi et al^9^ studied the effects of smoking on AC joint outcomes and found, also at 6-month follow-up, that coracoclavicular distance was significantly increased in patients who smoke compared to nonsmokers. While these two recent studies shed light on risk factors and AEs for AC joint injury, they are nonholistic in nature and do not account for short-term immediate indicators. Therefore, this study evaluates short-term, database-archived outcomes following AC joint surgeries for the purposes of enhancing patient education, outlining complication profiles, and supplementing updated surgical decision-making, hypothesizing that common comorbidities confer risks in alignment with prior literature.

## Methods

### Database

In this retrospective database study, the American College of Surgeons National Surgery Quality Improvement Program (NSQIP) database was queried to identify relevant cases of AC surgery. Based in the United States, the NSQIP database prospectively collects short-term (30-day) surgical data from participating institutions, with variables including preoperative characteristics, intraoperative variables, and rates of complications. In the most recent NSQIP database iteration (2023), a total of 994,313 cases were submitted across 676 participating sites and includes 274 unique variables.^41^ This database has demonstrated excellent reliability, with random audits of participating institutions finding inter-rater disagreement rates of approximately 2%.^10^^,^^40^

### Patient cohort

Patients who underwent AC joint surgery between 2010 and 2023 were identified. In this study, included cases designated Current Procedural Terminology 23550 [open treatment of AC dislocation, acute or chronic], 23552 [open treatment of AC dislocation, acute or chronic; with fascial graft (includes obtaining graft)], or 23120 [claviculectomy; partial] as either the primary or additional procedures. Cases were excluded if they had unknown or null values for sex, BMI, functional status, American Society of Anesthesiologists (ASA) classification, operative time, or anesthesia technique.

### Outcome metrics

In addition to demographic-related patient information, postoperative outcome data were collected for each patient and included surgical site infection, wound dehiscence, acute renal failure requiring dialysis, sepsis, pulmonary embolism, myocardial infarction, cardiac arrest, unplanned intubation, postoperative transfusion, deep vein thrombosis, pneumonia, urinary tract infection, return to operation room (ROR), death within 30 days, and any adverse event (AAE). AAE included any occurrence of surgical site infection, wound dehiscence, sepsis, unplanned intubation, postoperative transfusion, pneumonia, deep vein thrombosis, pulmonary embolism, urinary tract infection, cerebrovascular accident/stroke, cardiac arrest, myocardial infarction, congestive heart failure, ROR, and death within 30 days.

### Statistical analysis

Statistical analysis was performed using RStudio software version 2023.06.1 + 524 (R Foundation for Statistical Computing, Vienna, Austria). Metrics were described by mean (standard deviation) for continuous variables and number (%) for binary variables. To assess risk of adverse complications, multivariate odds-ratio logistic regression analysis was employed to model not only the associations between risk factors and marginal probabilities for binary outcomes, but also to model the associations between risk factors and pairwise dependency among binary outcomes in terms of odds ratios (OR). These risk factors were dynamically decided based on available comorbidities, with an ad hoc screening used to avoid model redundancy in correlated binary outcomes with high percent agreement values. Multivariate odds-ratio logistic regression was performed for risk of AAE. Separate multivariable analyses for low-incidence outcomes such as surgical site infection and ROR were explored post hoc due to clinical relevance and promising power; however, findings were inconclusive and thus not included due to insufficient event counts to support stable modeling and efficacious clinical statistics. In addition, a systematic threshold analysis was performed to identify the optimal operative time cut-point associated with increased complication risk. This approach evaluated multiple potential thresholds across the operative time distribution and identified the inflection point with the strongest association with AEs, as determined by maximizing the adjusted odds ratio while maintaining statistical significance (*P* < .05). Statistical significance was defined as *P* < .05.

## Results

### Demographic information

A total of 13,117 patients were identified as having underwent AC joint reconstruction between 2010 and 2023. The average age was 49.6 ± 15.2 years, average BMI was 30.1 ± 6.44 kg/m^2^, 21.4% of patients were smokers, 8.3% had non–insulin-dependent, or type 2 diabetes, and 2.9% had a history of chronic obstructive pulmonary disease (COPD). Most patients in the cohort were male (70%), and 24.9% were American Society of Anesthesiologists (ASA) class III. A detailed outline of patient demographic-related information is provided in Table I.

**Table I:** Demographic and comorbidity-related information for patients who underwent AC joint surgery between 2010 and 2023.

Metric	Mean (SD) or n (%)
Patients (n)	13,117
Age (yr)	49.6 (15.2)
Sex = male	9,244 (70.5%)
Body mass index (kg/m^2^)	30.1 (6.44)
Inpatient/outpatient = outpatient	11,780 (89.8%)
Smoker = yes	2,807 (21.4%)
Functional status = independent	13,065 (99.6%)
Functional status = partially dependent	47 (0.4%)
Functional status = totally dependent	5 (0.0%)
Dialysis required = yes	27 (0.2%)
Steroid use = yes	222 (1.7%)
Bleeding disorder = yes	138 (1.1%)
Ascites = yes	3 (0.0%)
Diabetes = insulin	494 (3.8%)
Diabetes = noninsulin	1,093 (8.3%)
COPD history = yes	378 (2.9%)
CHF history = yes	52 (0.4%)
Hypertension medication = yes	4,449 (33.9%)
Race = American Indian or Alaska Native	221 (1.7%)
Race = Asian	198 (1.5%)
Race = Black or African American	1,012 (7.7%)
Race = unknown/not reported	1,990 (15.2%)
Race = White	9,478 (72.3%)
ASA classification (categorical) = 1-no disturb	2,203 (16.8%)
ASA classification (categorical) = 2-mild disturb	7,503 (57.2%)
ASA classification (categorical) = 3-severe disturb	3,260 (24.9%)
ASA classification (categorical) = 4-life threat	149 (1.1%)
ASA classification (categorical) = 5-moribund	2 (0.0%)

*AC*, acromioclavicular; *SD*, standard deviation; *CHF*, congestive heart failure; *COPD*, chronic obstructive pulmonary disease; *ASA*, American Society of Anesthesiologists.

### Perioperative data and postoperative complications

The overall rate of developing any short-term postoperative complication/AE was low (2.7%). From this, surgical site infection (1.2%) and ROR (1.0%) rates were also low. A complete list of perioperative and postoperative complication-related information is provided in Table II.

**Table II:** Operative metrics and postoperative complication rates in patients who underwent AC joint surgery between 2010 and 2023.

Metric	Mean (SD) or n (%)
Operative time (min)	85 (56)
Blood transfusion = yes	7 (0.1%)
Surgical Site Infection = 1	155 (1.2%)
Death = 1	11 (0.1%)
Renal complication = 1	4 (0.0%)
Sepsis = 1	34 (0.3%)
Intubation required = 1	20 (0.2%)
Transfusion = 1	50 (0.4%)
Pneumonia = 1	31 (0.2%)
Urinary tract infection = 1	17 (0.1%)
Cardiac arrest = 1	2 (0.0%)
Deep vein thrombosis = 1	32 (0.2%)
ROR = 1	132 (1.0%)
Any adverse event = 1	348 (2.7%)
Wound dehiscence = 1	23 (0.2%)
Pulmonary embolism = 1	22 (0.2%)
CVA/stroke = 1	1 (0.0%)
Myocardial infarction = 1	8 (0.1%)

*AC*, acromioclavicular; *SD*, standard deviation; *CVA*, cerebrovascular accident; *ROR*, return to operation room.

Regarding multivariate odds-ratio logistic regression analysis for risk of AAE, operative time (OR = 1.01 [1.00-1.01]; *P* < .001), history of COPD (OR = 2.47 [1.55-3.81]; *P* < .001), history of steroid use (OR = 3.16 [1.82-5.18]; *P* < .001), dialysis (OR = 5.57 [1.86-14.99]; *P* = .001), bleeding disorder (OR = 2.67 [1.39-4.78]; *P* = .002), and insulin-dependent, or type 1 diabetes (T1D) (OR = 1.61 [1.05-2.52]; *P* = .044) demonstrated increased risk of developing at least one postoperative complication. Odds-ratio results for all studied comorbidities and operative metrics pertaining to adverse complication risk are provided in Table III.

**Table III:** Multivariate odds-ratio logistic regression for risk of AAE.

Metric	Odds ratio	Confidence interval	*P* value
Age (yr)	1	(0.99-1.01)	.718
BMI (kg/m^2^)	1	(0.98-1.02)	.882
Operative time (min)	1.01	(1.00-1.01)	<.001
Smoking history	1.25	(0.95-1.64)	.100
COPD history	2.47	(1.55-3.81)	<.001
Hypertension medication history	1.09	(0.82-1.44)	.555
Steroid history	3.16	(1.82-5.18)	<.001
CHF history	2.3	(0.78-5.68)	.096
Dialysis	5.57	(1.86-14.99)	.001
Bleeding disorder	2.67	(1.39-4.78)	.002
Ascites	10.68	(0.88-256.05)	.071
Intraoperative transfusion	7.75	(1.19-66.01)	.038
Diabetes (insulin-dependent)	1.61	(1.05-2.52)	.044
Diabetes (noninsulin-dependent)	1.24	(0.83-1.82)	.275

*AAE*, any adverse event; *BMI*, body mass index; *CHF*, congestive heart failure; *COPD*, chronic obstructive pulmonary disease.

### Operative time threshold analysis

**Figure 1:**
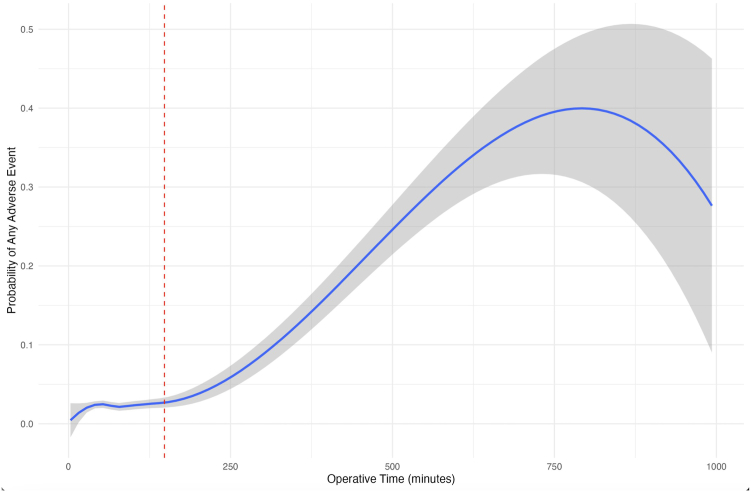
Relationship between operative time (minutes) and the probability of AAE. The *red, dashed line* indicates the identified threshold of 148 minutes, with the *blue line* indicating the mean probability, and the *gray* region indicating the 95% confidence interval. *AAE*, any adverse event.

With increased operative time demonstrating slightly increased risk of developing at least one postoperative complication per multivariate analysis calculations, and recognizing that the relatively smaller primary model OR described above was likely influenced by the wide distribution within the dataset, threshold testing was utilized to determine the ideal cut-off time needed to limit complication risk. An operative cut-off time of 148 minutes was determined: patients below this threshold experienced a 2.3% rate of developing at least one postoperative complication, and patients above this threshold experienced a 5.8% rate (OR = 2.67 [2.04-3.47]; *P* < .001). The relationship between operative time and the likelihood of experiencing an adverse postoperative event is shown in Figure 1.

## Discussion

This study utilized database-archived short-term results of AC joint surgeries to describe a broader view on this pathology beyond the limited prior data that commonly focused on outcomes such as reoperations and structural failures.^6^^,^^11^ While AC joint surgeries have been well-documented to carry a measurable risk of postoperative complications, the present study evaluated over 13,000 patients' short-term outcomes across a decade of data allocation, offering one of the largest analyses of surgical correction for AC joint injury to date, exceeding prior large sample sizes by more than 600%.^14^ Such a scale of analysis strengthens this study's contribution towards addressing a longstanding gap in existing literature.

Infections are among the most frequently reported complications following AC joint surgeries, as previously described by Gowd et al^14^ at a rate of 6.3%. In contrast, the present study found a much lower short-term infection rate of 1.2%, a finding that is both supported and challenged by existing literature. Marcheggiani et al^25^ reported a long-term infection rate of 2.3% following AC joint surgery, while other studies have found shorter-term rates of 5.8% and 2.4%, contextualizing our findings within the broader range of documented outcomes.^29^^,^^16^ As many of these studies often involve smaller sample sizes and record infections beyond 30 days postoperatively, direct comparison is challenging; nevertheless, the present study demonstrated that short-term infection risk is modest. Given the ongoing advances in surgical techniques and infection control in medicine, it is reasonable to presume that the comparatively lower rate of infections observed reflects recent improvements in orthopedic care; however, future root-cause analyses are warranted to identify more granular drivers.

Similarly, ROR was also observed at an acceptably low rate of 1.0% in the short term. This finding understandably contrasts the available longer-term literature, where overall reoperation rates for AC joint surgery are reported to be around 10% within a 6-month postoperative window.^6^^,^^38^ Interestingly, prior work has selectively queried ROR for AC joint irrigation and debridement within 30 days and found a 2.6% rate, while cumulative revision-specific AC reoperations within 6 months have been reported at a 4.2% rate.^43^ These comparative percentages of discrete reoperation causes, observed within short- to mid-term follow-ups, help to contextualize and partially bracket the 30-day findings of the present study. Accordingly, the higher overall reoperation rates (∼10%) described above in prior literature likely reflect the accumulation of major reoperations, such as revisions, alongside minor procedures over recovery timelines extending even into the long-term. While the root cause of reoperations in our study could not be determined due to NSQIP's granularity limitations, collating minor and major reoperation profiles across the postoperative course may help clarify how the larger overall reoperation rates reported in AC surgery literature are formed. Future studies should aim to include more stratified and detailed perioperative data to better isolate specific contributors to reoperations.

Multivariate logistic regression analysis revealed several insights concerning factors associated with the occurrence of AAE in AC joint surgery. The risk of experiencing AAE was significantly increased with longer operative times. This relationship remains debated in existing literature, as a study by Igrek et al^20^ reported that although arthroscopy assisted AC joint surgery techniques had longer operative times than percutaneous approaches (average 61.1 vs. 34.7 minutes; *P* = .001, respectively), the percutaneous group experienced more structural complications, presumed by the authors to be due to suboptimal tunnel placement. In contrast, Peng et al found that a single tunnel technique, with longer operative time compared to a coracoid sling technique (average 96.3 vs. 73.7; *P* < .01, respectively), was associated with higher complication rates (*P* < .01). This finding is supportive of the present study and is well-documented in orthopedic literature regarding operative time and complications.^8^^,^^30^ The aforementioned conflicts not only highlight the variety of operative times associated with the myriad of AC joint surgery techniques, but also emphasize the notion that increased complication risk due to prolonged operative time, such as in arthroscopic interventions, may be tied to the technique of choice and should be considered during shared decision-making.^1^ While the NSQIP database reported a mean operative time of 85 ± 56 minutes for a composite of AC joint surgeries, it did not specify the frequencies of the individual procedures reflected in the standard deviation, and this must be considered when interpreting this study's findings. Importantly, the present study identified a 148-minute threshold as the point at which the highest discernible odds ratio at statistical significance occurs, which is a clinically more meaningful estimate given that the primary operative time model was likely overpowered. Such findings further clarify the role of operative time as a crucial risk factor when contextualized appropriately.

Several comorbidities were found to significantly increase the risk of experiencing AAE. As previously noted, COPD was associated with a significantly increased risk of AAE. While literature on this topic specific to AC joint injuries is limited, COPD has been recognized as a strong predictor of pulmonary complications postoperatively in orthopedic literature, a finding that may be due to surgical anesthesia and the resultant minor cases of atelectasis and ventilation/perfusion mismatch.^15^^,^^28^ Steroid history was also associated with a significantly increased risk of AAE; however, literature support on this comorbidity is mixed. Huang et al^18^ reported no significant differences in complications following rotator cuff repairs in patients with autoimmune connective tissue diseases who used steroids vs. controls. Contrastingly, Forsythe et al, in a large database study, found higher complication rates in patients undergoing rotator cuff repair who received corticosteroid injections within one month of surgery, a sequence of events that may be analogous to AC joint injury practices.^12^^,^^17^ Although systemic steroid use has been linked to poor healing, the aforementioned conflicting findings elucidate the need for more focused research on this topic with respect to AC joint surgeries to supplement the findings of the present study.^19^

Though limited to 27 cases, the present study found that patients with end stage renal disease requiring dialysis had a significantly increased risk of experiencing AAE following AC joint surgery. Although direct evidence in AC joint procedures is limited, NSQIP-based studies on rotator cuff repairs and knee arthroscopies have similarly reported dialysis as a significant risk factor for experiencing AAE, reinforcing its relevance as a well-documented comorbidity in orthopedic literature.^4^^,^^23^ Tandemly, bleeding disorders were also linked to a significantly increased risk of experiencing AAE. NSQIP-based studies on rotator cuff repairs also reported this association, presumably due to the increased risk of postoperative transfusions, sepsis, hospital admission, or mortality.^2^ Lastly, T1D was also a comorbidity associated with a significantly increased risk for experiencing AAE. Prior studies across shoulder and other orthopedic surgeries have demonstrated increased rates of complications in T1D patients, including infections and joint stiffness, prompting evidence-based recommendations for surgeon caution and vigilance when counseling and treating this patient population.^7^^,^^28^

When interpreting the present study's NSQIP-based findings, several limitations must be considered. Most notably, the NSQIP database only captures outcomes within 30 days postoperatively, inherently missing long-term complications such as structural failures. In addition, neither the database nor the Current Procedural Terminology coding options differentiate between graft type or surgical techniques, such as open-only vs. arthroscopy-utilizing AC joint surgery, limiting the ability to draw procedure-specific insights that may greatly impact postoperative clinical outcome profiles. Furthermore, comorbidities are recorded as binary variables, which oversimplifies their clinical impact as conditions existing on spectrums that naturally influence disease progression and informed decision-making for surgery. For instance, the NSQIP database does not distinguish between a short-term or long-term steroid or insulin user, adding difficulty to the assessment of temporal and cumulative effects of such medications on short-term outcomes. This lack of data granularity makes more nuanced analysis of outcomes challenging. Other important variables, including time to surgery, institutional case-volume for experience assessment, injury history, and injury severity measures like Rockwood classification, are not captured in NSQIP: all factors that could have substantially influenced outcomes, supplemented analyses, and strengthened conclusions. Considering such constraints, future research is needed with more granular clinical data, such as through stratification of outcomes by surgical technique, graft selection, and severity classification, to better delineate outcomes, risk profiles, and potential root-causes. Nonetheless, the present study offers one of the largest sample sizes to date on AC joint surgery to address a critical gap in the literature by casting a wide net across a broad patient population and describing the resultant outcomes.

## Conclusion

This short-term NSQIP database study demonstrated reassuring outcomes for AC joint surgeries, with acceptably low rates of postoperative complications. Notably, complications related to infections and ROR were relatively uncommon. Longer operative time was significantly associated with increased risks for AAE, with the highest risk observed beyond a 148-minute threshold, an indication that AC joint surgery operative time may need to be considered for optimal outcomes. Patients with a history of COPD, steroid use, dialysis, bleeding disorders, and T1D were all susceptible to increased risk of developing AAE. Due to this, perioperative patient counseling is crucial to further limit added risk for AC joint surgery AEs. Within the context of an inherently limited NSQIP database study, the overall short-term outcomes of AC joint surgeries demonstrated great results, with all complication rates at or below the rates described in the literature.^17^

## Disclaimers:

Funding: This study did not receive any support in the form of grants, equipment, or other resources.

Conflicts of interest: The authors, their immediate families, and any research foundation with which they are affiliated have not received any financial payments or other benefits from any commercial entity related to the subject of this article.
